# Biology, Ecology, and Management of Prevalent Thrips Species (Thysanoptera: Thripidae) Impacting Blueberry Production in the Southeastern United States

**DOI:** 10.3390/insects16070653

**Published:** 2025-06-24

**Authors:** Rosan Adhikari, David G. Riley, Rajagopalbabu Srinivasan, Mark Abney, Cera Jones, Ashfaq A. Sial

**Affiliations:** 1 Department of Entomology, University of Georgia, Athens, GA 30602, USA; ra84320@uga.edu (R.A.); cera@uga.edu (C.J.); 2 Department of Entomology, University of Georgia, Tifton, GA 31794, USA; dgr@uga.edu (D.G.R.); mrabney@uga.edu (M.A.); 3 Department of Entomology, University of Georgia, Griffin, GA 30223, USA; babusri@uga.edu

**Keywords:** berry crop, *Frankliniella tritici*, *Frankliniella bispinosa*, *Scirtothrips dorsalis*, IPM

## Abstract

Blueberry is a high-value fruit crop in the United States, with Georgia and Florida serving as important early-season production regions. In these areas, several thrips species (Thysanoptera: Thripidae), including *Frankliniella tritici* (Fitch), *Frankliniella bispinosa* (Morgan), and *Scirtothrips dorsalis* (Hood), have emerged as economically significant pests. While *F. tritici* and *F. bispinosa* primarily damage floral tissues, *S. dorsalis* targets young foliage. Their rapid reproduction, high mobility, and broad host range contribute to rapid population buildup and complicate the management programs. Species identification is often difficult due to overlapping morphological features and requires the use of molecular diagnostic tools for accurate identification. Although action thresholds, such as 2–6 *F. tritici* per flower cluster, are used to guide management decisions, robust economic thresholds based on yield loss remain undeveloped. Integrated pest management (IPM) practices include regular monitoring, cultural control (e.g., pruning, reflective mulch), biological control using *Orius insidiosus* (Say) and predatory mites, and chemical control. Reduced-risk insecticides like spinetoram and spinosad offer effective suppression while minimizing harm to pollinators and beneficial insects. However, the brief flowering period limits the establishment of biological control agents. Developing species-specific economic thresholds and phenology-based IPM strategies is critical for effective and sustainable thrips management in blueberry cropping systems.

## Simple Summary

Blueberry is a popular fruit known for its health benefits, including high levels of vitamins, antioxidants, and anti-inflammatory compounds. As demand for blueberries has grown, global production has more than doubled in the past decade. In the United States, Georgia and Florida are leading producers of early-season blueberries, with harvests beginning as early as April. Nevertheless, growers in these regions face increasing challenges from many insect pests, including thrips. These pests damage blueberry flowers and leaves by feeding and laying eggs, which can reduce fruit quality and yield. This paper focuses on the biology, movement, and feeding behavior of the most common thrips species found in southeastern blueberry production systems, as well as the environmental factors that influence their populations. It also reviews current pest control methods, including monitoring, cultural practices like pruning and reflective mulch, biological control agents, and the use of safer insecticides. Understanding how these pests behave and how to manage them is important for helping growers produce healthier blueberries while reducing crop losses and minimizing harm to pollinators and the environment.

## 1. Introduction

*Vaccinium* is a common and widespread genus of berry-producing shrubs in the Ericaceae family. It consists of approximately 450 species widely distributed in the northern hemisphere, tropical Asia, and Central and South America [1]. Many species in this genus are consumed by humans, and some, including cranberry, blueberry, bilberry, lingonberry, and huckleberry, hold significant commercial value [2]. Blueberries are renowned as a superfood, rich in vitamins, minerals, and antioxidants such as flavonoids and phenolic acids. Their health benefits, especially related to antioxidants and anti-inflammatory properties, are largely due to their rich content of polyphenols, particularly the anthocyanin pigment [3].

Driven by growing consumer demand for their nutritional benefits, global production of blueberries increased from 439,000 metric tons to nearly 1 million metric tons between 2010 and 2019 [4]. The prominent blueberry-producing countries are the United States, Canada, Chile, and France. In the United States, blueberries are the second-most-produced berry [5] with the prominent states being California, Florida, Georgia, Michigan, New Jersey, North Carolina, Oregon, and Washington. In 2023, the farm gate value of blueberry production in Georgia was USD 526.6 million [6]. Georgia and Florida are the primary U.S. states that produce a significant quantity of early-season blueberries, harvesting the crop in April and May [7].

As production has intensified, particularly in early-season regions like Georgia and Florida, the risk of pest outbreaks has also increased. Several arthropod species are considered economically important pests of blueberry in Georgia and Florida. These arthropod species include, but are not limited to, spotted-wing drosophila (SWD), *Drosophila suzukii* Matsumura, thrips, blueberry gall midge, *Dasineura oxycoccana* Johnson, blueberry bud mite, *Acalitus vaccinia* Keifer, and blueberry maggot, *Rhagoletis mendax* Curran. In recent years, thrips have emerged as one of the major pests causing significant damage to blueberry production [8,9]. Thrips are omnipresent, slender-bodied insects whose size is usually less than 2 mm [10]. The damage caused by the thrips species in agricultural commodities is primarily due to feeding and oviposition on the leaves, flowers, and fruits [11].

## 2. Thrips in Blueberries

A diverse complex of thrips species has been reported infesting blueberry crops across North America, with notable geographic variation. In total, at least 10 thrips species have been documented on blueberries in the United States. These include *F. tritici*, *F. bispinosa*, *Frankliniella occidentalis*, *Frankliniella fusca*, *Frankliniella hawaiiensis*, *Frankliniella vaccinii*, *Scirtothrips ruthveni*, *Scirtothrips citri*, *S. dorsalis*, and two *Catinathrips* species (*C. vaccinophilus* and *C. kainos*) [12,13,14,15,16]. Among these, *F. tritici* (flower thrips), *F. bispinosa* (Florida flower thrips), and *S. dorsalis* (chilli thrips) are considered the most economically significant pests of cultivated blueberries [17]. These species are pests of rabbiteye and southern highbush blueberries in the southeastern United States [18,19]. In Florida, the predominant species is *F. bispinosa*, making up 83.6% of the thrips collected on sticky traps in blueberry fields. This species is known to cause considerable economic damage by feeding injury to various crops, including citrus, blueberries, strawberries, and field peppers [20]. Conversely, in Georgia, *F. tritici* is the most common species, accounting for 94.0% of the thrips captured [7,16]. *Scirtothrips dorsalis* was first recorded in 2008 on blueberries in several counties across Florida [18]. Since then, it has become an increasingly problematic pest for blueberry growers. Other species found in southeastern U.S. blueberry crops include *Frankliniella fusca* (Hinds), tobacco thrips, *Frankliniella occidentalis* (Pergande), western flower thrips, and *Frankliniella hawaiiensis* (Morgan) [16]. These species represent a very small percentage (less than 5%) of the total thrips population and, therefore, are not discussed in this review.

The major symptoms of *F. tritici* and *F. bispinosa* injury are leaf curling and malformation, with injury to the styles and green tissues in flowers [21]. In blueberries, they are a significant early-season pest also causing injury by feeding on the ovaries, styles, petals, and developing fruits [16,22]. In contrast, *S. dorsalis* typically feeds mainly on the fresh vegetative parts of the blueberry plants following summer pruning [18]. These three species are known for their wide host range and can cause extensive damage to various crop plants.

Identification of *Frankliniella* species is often complicated due to their similar morphological characteristics, especially in field conditions where multiple species coexist [20]. Shared traits such as body size and coloration make it difficult to distinguish between species without high magnification and close examination of key diagnostic features [20]. In many cases, accurate field identification is nearly impossible without expert assistance [20]. Ideally, a combination of morphological and molecular approaches should be used for reliable species identification. Morphological identification typically involves collecting thrips specimens, slide mounting them, and examining them under a microscope, and this process is particularly critical when working with members of the *Frankliniella* genus [23]. However, when morphological identification becomes very difficult, time-consuming, or inconclusive, molecular methods like DNA barcoding provide a valuable and dependable alternative for accurate species determination [24].

## 3. Biology of Thrips

The typical life cycle of thrips species found in the blueberry production system is similar to other members of the Thripidae family. It includes an egg stage, two actively feeding larval stages, followed by two relatively inactive pupal stages, and finally, the adult stage [25]. Using their saw-like ovipositor, adult female thrips insert eggs into various plant parts, including leaves, petioles, flower bracts, petals, and developing fruit [26]. The duration of incubation and development in thrips varies by species and is influenced by environmental conditions [27]. Eggs typically hatch within a range of 2 to 26 days, with the exact timing influenced by temperature and humidity [27,28,29,30,31]. Specifically, eggs hatch most efficiently at an optimum temperature of 23–25 °C and relative humidity of 65–70%, which supports faster embryonic development and shorter hatching periods [32]. After hatching, plant-feeding thrips larvae emerge and develop over a period of 2 to 13 days, depending on species and environmental conditions [27,28,29,30,31]. This larval development is followed by a prepupal stage, which is mobile but does not involve feeding. Approximately 1 to 5 days later, the larva leaves the plant and enters the soil to pupate. The pupal stage lasts between 1 and 10 days, after which the adult thrips emerge from the soil [27,31].

Among the thrips species found in blueberry systems, *F. tritici* has received limited research attention compared to the extensively studied *F. occidentalis.* Despite broad similarities in development and reproduction between *F. occidentalis* and *F. tritici*, variations in temperature can differentially impact their developmental periods [33]. Like many other thrips, *F. tritici* follows a haplodiploid reproductive system, producing males from unfertilized eggs and females from fertilized ones [33]. Watts (1936) noted that at 28 °C, *F. tritici* had a slightly faster development than *F. occidentalis*, with a median immature development time shorter by one day [30]. On average, the life cycle of *F. tritici* requires 15.01 days, females lay 41.25 eggs during their lifetime, and adults live 21.42 days [30]. The mean daily fecundity of *F. tritici* was over 9% higher compared to that of *F. occidentalis* [33]. This reproductive advantage, along with its relatively faster development time, may contribute to more rapid population growth in *F. tritici* compared to *F. occidentalis*. Additionally, *F. occidentalis* experiences substantial interspecific competition from *F. tritici*, while *F. tritici* appears unaffected by the presence of *F. occidentalis* [34]. These factors collectively may explain the greater abundance of *F. tritici* in the southeastern United States [35].

Another important species of thrips found in blueberries is *F. bispinosa*, which displays a temperature-dependent life cycle that is comparable to *F. tritici*. At lower temperatures (15 °C), it takes approximately 37.5 ± 1.35 days to complete development, with adult females living up to 30 ± 5.8 days. However, at higher temperatures (35 °C), the immature development period and adult female longevity are drastically reduced to just 9.2 and 3.9 days due to stress from high temperature, respectively. Fecundity of *F. bispinosa* is at its maximum at 25 °C, at which females lay an average of 123.2 ± 22.2 eggs, which is significantly higher than at 15 °C (29.5 ± 10.9 eggs), 20 °C (42.1 ± 9.1 eggs), or 30 °C (11.6 ± 4.4 eggs) [36]. Adult *F. bispinosa* also outcompetes *F. occidentalis* in their ability to move more quickly in field peppers than *F. occidentalis*, which could help them evade predators more effectively [37].

*Scirtothrips dorsalis*, another species of emerging concern in southern blueberry systems, is notable for its rapid development and reproductive capacity under optimal environmental conditions. It has a relatively short life cycle under optimal temperatures of 25–27 °C, completing development from egg to adult in about 18–20 days [38]. The larval period lasts 8 to 10 days, during which larvae feed and grow rapidly. The pupal stage, lasting approximately 2.6 to 3.3 days at a temperature of 25–30 °C and relative humidity of 60–70%, occurs in protected microhabitats such as curled leaves, leaf axils, under flower calyces, or in leaf litter [15]. Environmental conditions influence the duration of each stage, with the total life cycle ranging from 14 to 20 days. A single female can lay between 60 and 200 eggs in her lifetime, contributing significantly to rapid population buildup [39]. Together, these three species (*F. tritici*, *F. bispinosa*, and *S. dorsalis*) represent the most economically important thrips pests in southeastern U.S. blueberry systems. While they share a common general life cycle structure, species-specific differences in development time, fecundity, and thermal tolerance have important implications for population dynamics and management strategies in commercial blueberry production.

## 4. Host Range of Thrips

Thrips are highly polyphagous insects and can be found on a wide range of crop and weed species. Many of these alternative host plants are commonly present within and around blueberry production systems, providing thrips with abundant food sources and breeding sites. These alternative hosts serve both as feeding and reproductive sites, supporting thrips survival during periods when blueberries are not in bloom. As a result, they contribute to the buildup of thrips populations, which can later migrate to blueberry plants and cause damage. Thrips are also highly mobile insects, with a strong preference for floral tissues, where they feed, reproduce, and complete their life cycle [40]. Their ability to move readily between plants in response to floral availability allows them to migrate from nearby vegetation into cropping systems; migrations often align with the flowering periods of wild or cultivated hosts [20]. Moreover, adult thrips can travel long distances by riding air currents, which means that the thrips observed at a given location may have migrated from nearby or distant crops [41,42].

In blueberry-growing regions of the southeastern United States, *F. tritici*, *F. bispinosa*, and *S. dorsalis* are the most prevalent species, and each exhibits unique host associations and seasonal dynamics. A survey conducted by Chellemi et al. (1994) in 37 wild plant species, identified *F. tritici* as the most abundant species during the months of March, May, and August. In contrast, *F. bispinosa* was the predominant species in June and July. Meanwhile, *F. occidentalis* was found to be the most abundant in February and April. The peak populations of *Frankliniella* species coincided with the peak flowering period of wild host plants, occurring between March and June in the same geographical area. This alignment suggests that the abundance of these thrips species is closely linked to the availability of flowering wild host plants, highlighting the importance of floral resources in their population dynamics and the differing ecological preferences and life cycles of these thrips species throughout the year [43].

The presence and abundance of *F. tritici* during late winter and early spring have also been documented in northern Florida and Georgia. For example, adults of *F. tritici* were occasionally observed on vetch during winter, although it does not appear to serve as a preferred overwintering host [44]. As temperatures rose in spring, *F. tritici* populations became more abundant on vetch and clover. These trends align with earlier findings from Chamberlin et al. (1992) and Chellemi et al. (1994), which confirmed the winter presence of *F. tritici* in these regions [43,45]. Geographic differences also appear to influence thrips dynamics, as *F. tritici* has been reported to occur more abundantly in southern regions of the United States, with earlier colonization of soybean fields and higher overall population levels compared to northern areas [46,47].

*Frankliniella bispinosa’s* primary habitat is flowers; however, they can also feed on developing fruits, especially when their population density is high. *Frankliniella bispinosa* can rapidly reach high population levels, demonstrate strong adaptability, and maintain high densities year-round, making it a significant pest of many crops [36].

In contrast to *F. tritici* and *F. bispinosa*, *S. dorsalis* is considered an invasive species with an exceptionally broad host range, infesting over 100 plant species across more than 40 plant families [48]. This wide polyphagous behavior enables the species to exploit numerous plant types, contributing to its status as a significant agricultural pest. The adaptability of *S. dorsalis* to a variety of host plants increases its potential to inflict substantial economic damage, affecting key commercial crops such as fruits, vegetables, and ornamentals [49]. Environmental factors, especially temperature and solar radiation, play a pivotal role in regulating the abundance and behavior of *S. dorsalis* [50]. Thrips flight activity and reproductive patterns, including oviposition, are influenced by daily temperature fluctuations and light availability [50]. Research shows that their activity intensifies with increasing temperatures and higher solar radiation, which promotes flight and egg-laying behavior. In contrast, as temperatures decline and solar radiation diminish, the thrips reduce their activity levels, leading to a cessation in flight and reproduction [50].

The following sections provide detailed descriptions of these dominant species, highlighting their morphological characteristics, their impact on blueberry production, and their management in the blueberry production system.

## 5. Thrips Species Associated with Blueberry Flowers

### 5.1. Frankliniella tritici

In the eastern United States, *F. tritici* is one of the most abundant species among thrips [33]. *Frankliniella tritici* originates from eastern North America and is found globally, spanning regions such as Asia, the Caribbean, and Europe [51]. This thrips species is often confused with other *Frankliniella* species, such as *F. bispinosa* and *F. occidentalis*, both of which are known to effectively transmit several plant viruses in various crops, though no such transmission has been reported in blueberries [52].

**Figure 1:**
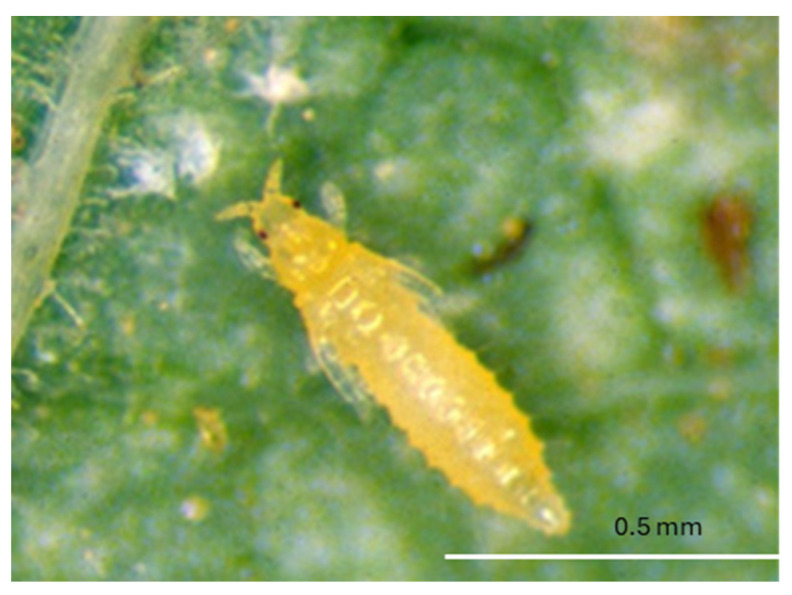
*Frankliniella tritici* larva (Picture credit: Stuart Reitz).

**Figure 2:**
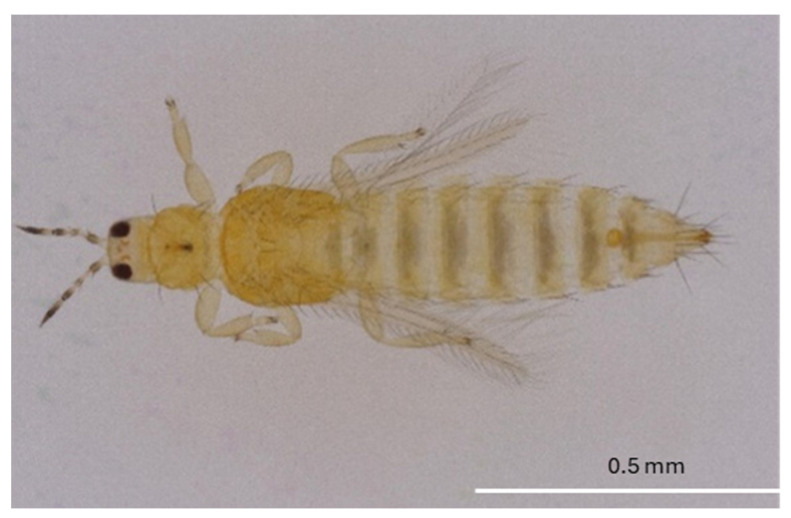
*Frankliniella tritici* adult (Picture credit: Rosan Adhikari).

The eggs of *F. tritici* are kidney-shaped and measure about 0.4 mm in length and 0.2 mm in diameter [53]. The larvae are wingless and yellow, resembling adults in appearance. These larvae are elongated, oval-shaped, and reach approximately 0.5 mm in length and 0.2 mm in diameter [51] (Figure 1). During the prepupal stage, the insect develops wing buds, and the antennae remain straight. However, in the pupal stage, which is 0.5–1 mm in length and the antennae are bent backward over the head [51]. Adults are elongated and approximately 1 mm in length [20] (Figure 2). Identification of adult *F. tritici* requires high magnification to identify key diagnostic features, which include the swollen pedicel on the third antennal segment, an interrupted microtrichial comb on the eighth abdominal segment, and the placement of the third ocellar setae, which does not arise between the posterior ocelli [51,53]. These morphological details, particularly at the adult stage, are crucial for differentiating *F. tritici* from other species within its genus.

### 5.2. Frankliniella bispinosa

*Frankliniella bispinosa* was first documented in Florida by Morgan in 1913 [54]. These thrips were consistently the dominant species in Florida and collected from more than 33 species of cultivated and non-cultivated plants [55]. In Florida, this thrips species was most abundant in the citrus groves and accounted for 80–100% of the specimens identified [56]. The first instar, second instar, and adults of this thrips species feed on the various parts of the citrus flowers and cause significant damage to the floral and bud tissues [56].

**Figure 3:**
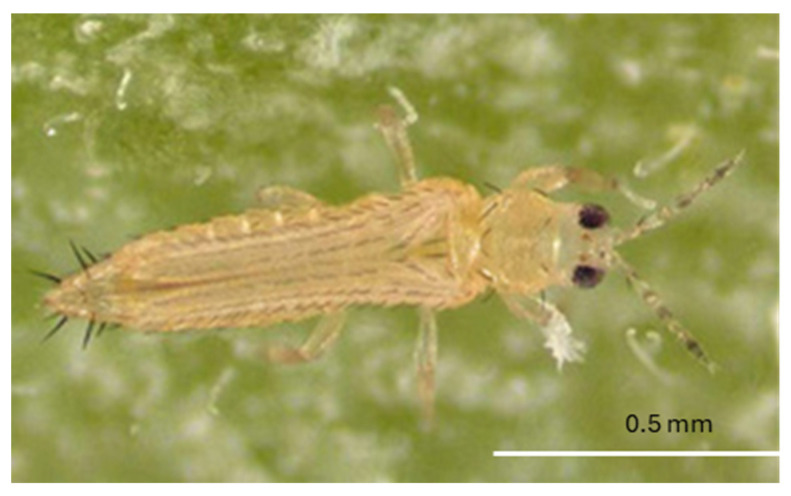
Adult *F. bispinosa* (Picture credit: Lyle Buss).

**Figure 4:**
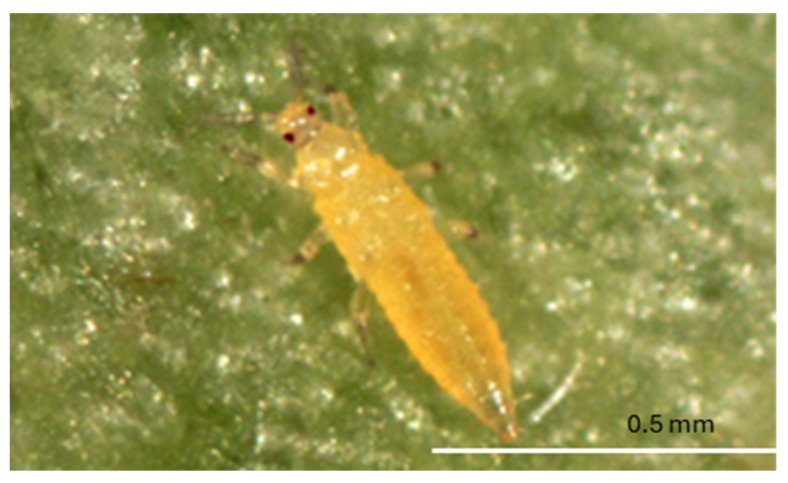
Larva of *F. bispinosa* (Picture credit: Jeff D. Cluever).

The body of adult *F. bispinosa* is slender and elongate, measuring around 1 mm in length, with females generally being slightly larger than males (Figure 3). The length and diameter of its stages are comparable to those of *F. tritici*. The larvae are yellow in color and elongate oval (Figure 4). Adult bodies and legs are predominantly yellow, adorned with brown setae (hairs), which contribute to their distinct appearance. The antennae are divided into eight segments, with the second segment featuring prominent brown spines [20]. This combination of physical traits, including small size, elongated body shape, and feathered wings, allows *F. bispinosa* to be highly mobile, moving easily between flowers and plants. *Frankliniella bispinosa* demonstrates strong adaptability and maintains high population density year-round and has become a significant pest of many crops [36]. *Frankliniella bispinosa* can rapidly reach high population levels, especially when predator populations have not yet been established. This species is known to cause considerable economic damage to various crops, including citrus, blueberries, strawberries, and field peppers [20].

### 5.3. Impacts of F. tritici and F. bispinosa on Blueberry

The greatest concentration of these thrips was found inside the canopy of blueberry bushes [7]. Aggregation of thrips known as hot spots was more likely to form in areas where more than seven thrips per day were caught on sticky traps, about 5 to 7 days after the bloom started [7]. These findings can help producers monitor thrips populations more effectively, allowing them to identify and manage hot spots before they escalate into more significant problems on blueberry farms [7]. The number of thrips inside blueberry flowers has been found to have a strong correlation with blueberry fruit injury, making it a more reliable sampling method compared to others [16].

**Figure 5:**
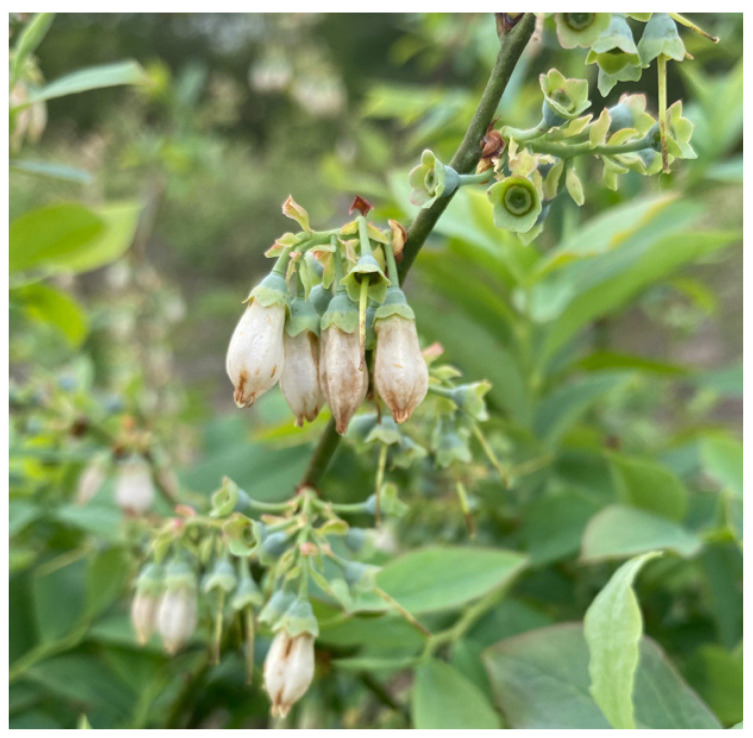
*Frankliniella tritici* injury to blueberry flowers (Picture credit: Rosan Adhikari).

**Figure 6:**
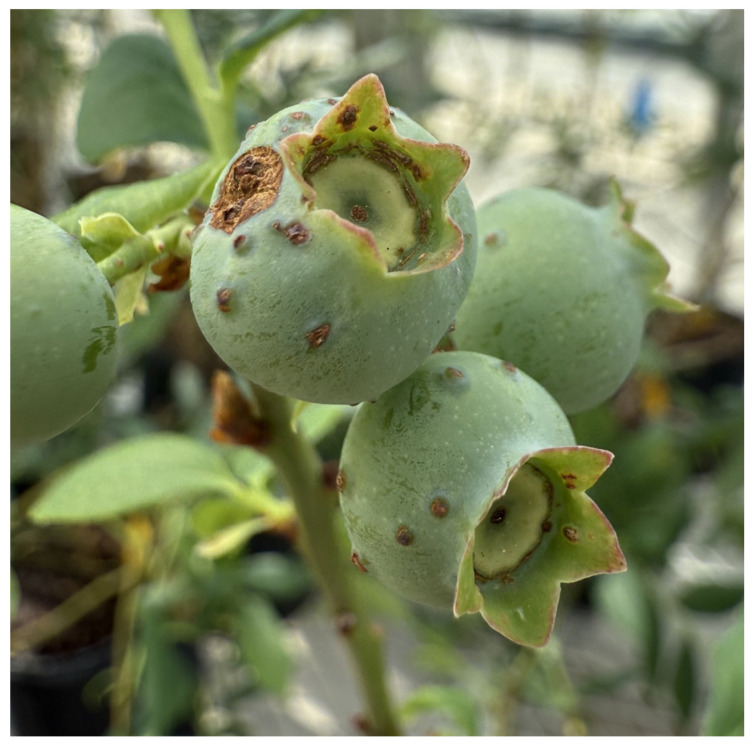
*Frankliniella tritici* injury to blueberry fruits (Picture credit: Rosan Adhikari).

Both *F. tritici* and *F. bispinosa* cause substantial damage to blueberry crops through similar mechanisms of injury. *Frankliniella tritici* primarily inhabits the interior of blueberry flowers, where adults and larvae inflict damage by feeding on the sap from flower tissues. This feeding targets vital reproductive structures within the flower, such as the petals, styles, ovaries, and developing fruit tissues [57] (Figure 5). As a result, the integrity of these tissues is compromised, impairing pollination and the development of viable fruit, which ultimately reduces yield and fruit quality [57] (Figure 6). In addition to their sap-feeding behavior, thrips may consume pollen, which can interfere with pollination and potentially lead to reduced fruit set or abortion. These feeding injuries, if extensive, can lead to significant yield reductions, posing a major threat to blueberry production [57].

Furthermore, female thrips of both species also lay their eggs within various parts of the flower tissues. The egg-laying process itself can leave scars, which may become visible on the mature fruit [33]. When the eggs hatch, the emerging larvae bore holes in the flower tissue, which can lead to malformed flowers and fruit, reducing the overall yield and marketability of the crop [58].

**Figure 7:**
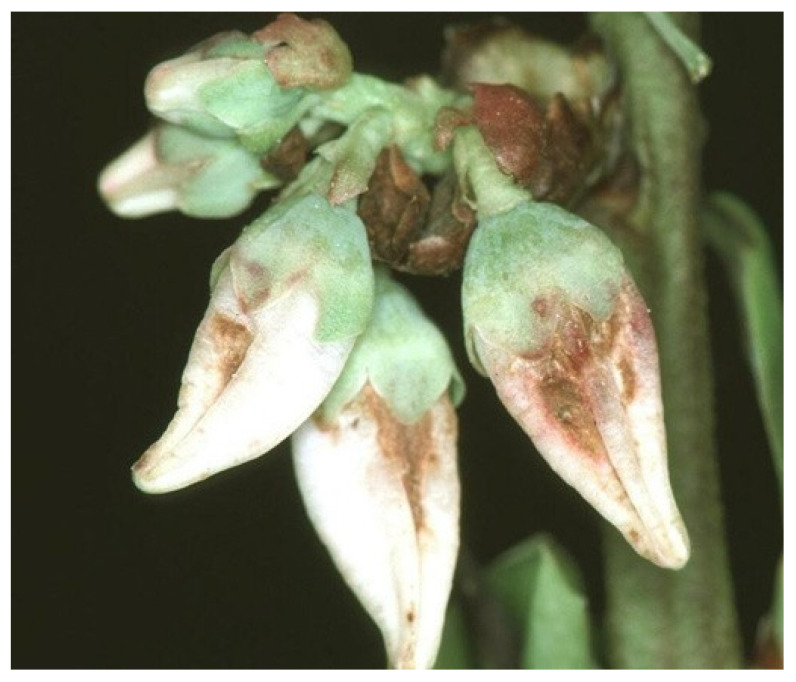
*Frankliniella bispinosa* injury to blueberry flowers (Picture credit: Jerry A. Payne).

**Figure 8:**
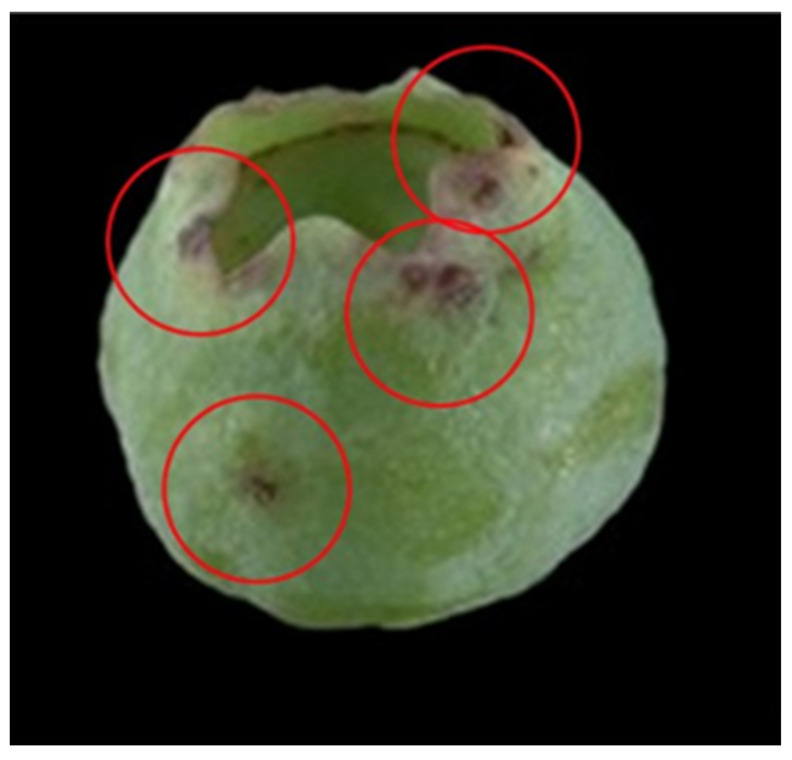
*Frankliniella bispinosa* injury to blueberry fruit, scars from egg laying, red circles showing feeding injury (Picture credit: H.A. Arévalo).

**Figure 9:**
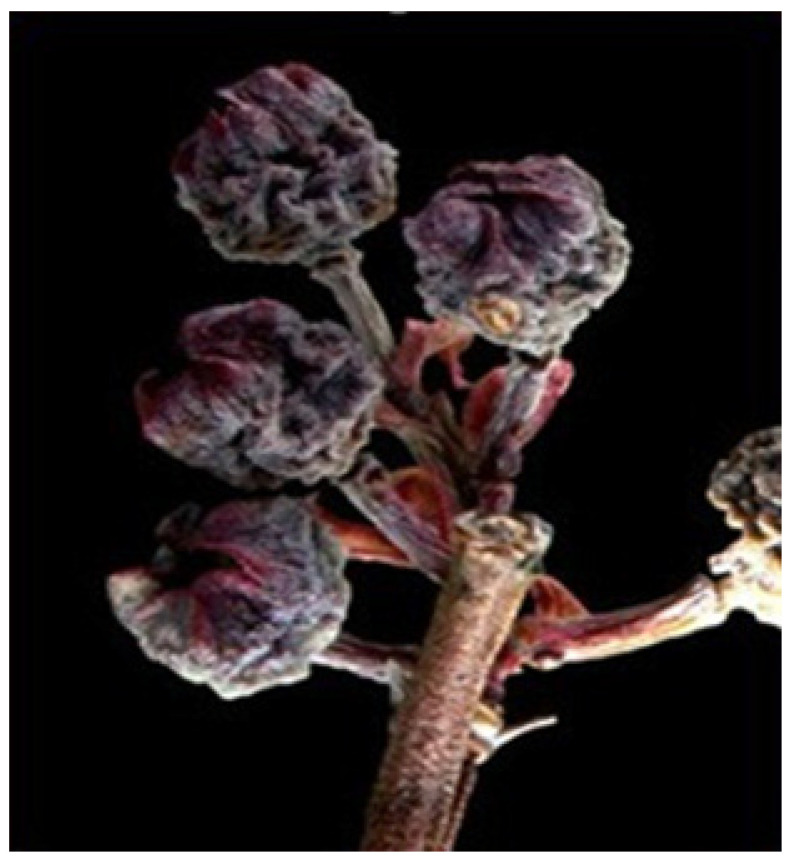
Fruit dehydration by 20 *F. bispinosa*/flower cluster (Picture credit: Oscar Liburd).

Like *F. tritici*, *F. bispinosa* infests a variety of crops and non-crop plants, including blueberries. Some blueberry growers in Florida have noticed that thrips populations in blueberry flowers tend to rise as citrus blooms begin to decline [16]. *Frankliniella bispinosa* causes damage to flowers through feeding and boring. These insects feed on the leaves, flowers, and fruit of blueberry plants (Figure 7, Figure 8 and Figure 9) [12]. The cumulative effect of feeding by both adult thrips and larvae, along with egg laying activities, can significantly impact the plant and its ability to produce high-quality fruit. *Frankliniella bispinosa* damage can also be identified by the tight curling and malformation of blueberry leaves. More critically, in addition to leaf injury, thrips prefer feeding on the style and the surrounding green tissue of flowers [12]. This feeding behavior can negatively impact pollination and reduce fruit set [58]. Polavarapu and Polk (2001) reported that *F. bispinosa* also consumes pollen and chlorophyll-rich green tissue within the flower. They can cause damage to the ovules, and in severe cases, this injury can lead to premature fruit drop, resulting in a direct loss of yield. High levels of infestation can cause visible pimpling on the fruit [12]. In Georgia, thrips populations have reached as high as 40–50 individuals per flower cluster, with a reduction in fruit set of up to 60% attributed to thrips damage in Southern highbush blueberries [12]. The visual damage symptoms shown in Figure 7, Figure 8 and Figure 9 represent a progression of injury severity, which can be used by growers to assess infestation levels and make timely management decisions.

## 6. Thrips Species Associated with Blueberry Foliage

### 6.1. Scirtothrips dorsalis

*Scirtothrips dorsalis* is a significant pest affecting vegetable, fruit, and ornamental crops across Asia, Africa, Oceania, the Caribbean, and parts of South America. They are also an invasive pest in several U.S. states [59]. *Scirtothrips dorsalis* were initially spotted in Florida in 1991 and again in 1994, but it was not until October 2005 that the first established population was documented on roses in Palm Beach County [18]. *Scirtothrips dorsalis* was first reported in Georgia in 2007 [39].

**Figure 10:**
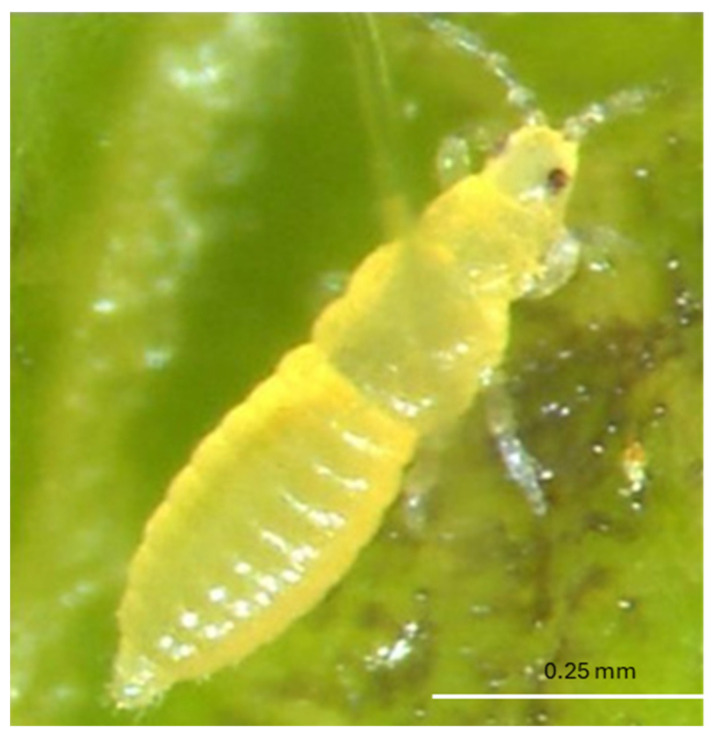
Larva of *S. dorsalis* (Picture credit: Babu Panthi).

**Figure 11:**
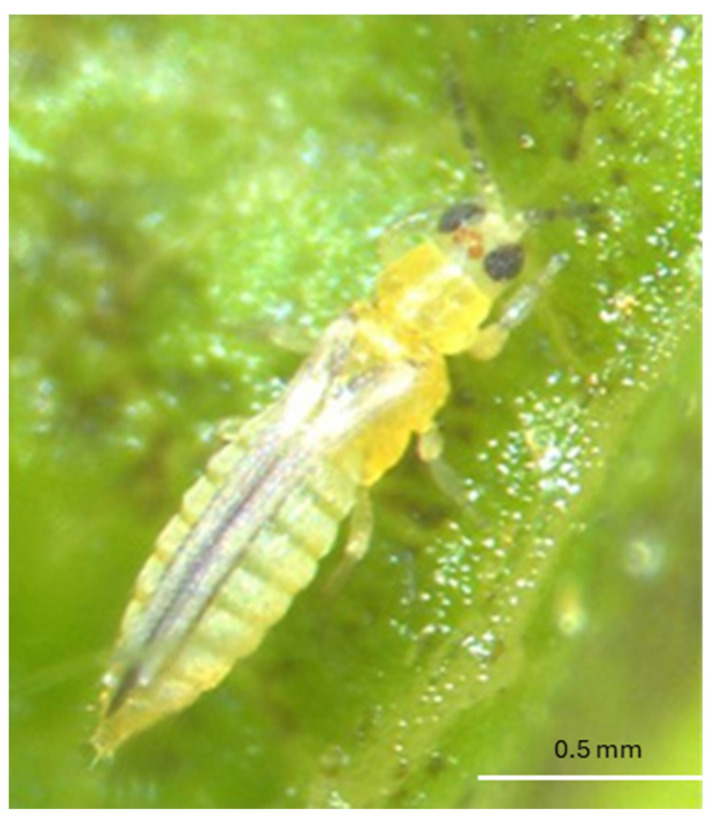
Adult female *S. dorsalis* (Picture credit: Babu Panthi).

*Scirtothrips dorsalis* is a small and fast-moving insect that presents challenges in detection when inspecting fresh vegetation [60]. The larvae of *S. dorsalis* are creamish white to pale in color [15] (Figure 10). The adults measure approximately 1.2 mm in length (Figure 11) and are distinguished by their dark wings and characteristic dark spots on the abdomen, forming incomplete stripes visible from a dorsal view. A key identification feature is the presence of a complete posteromarginal comb on the eighth abdominal segment. This species has eight antennal segments, with the first two (I and II) being pale, while the remaining segments (III through VIII) are darker in color. Additionally, antennal segments III and IV possess distinctive forked sense cones, which serve as an important morphological trait for identifying this thrips species [50,60]. *Scirtothrips dorsalis* is highly adaptable and can quickly establish when introduced to a new region, allowing it to thrive and spread [61].

### 6.2. Impacts of S. dorsalis on Blueberry

**Figure 12:**
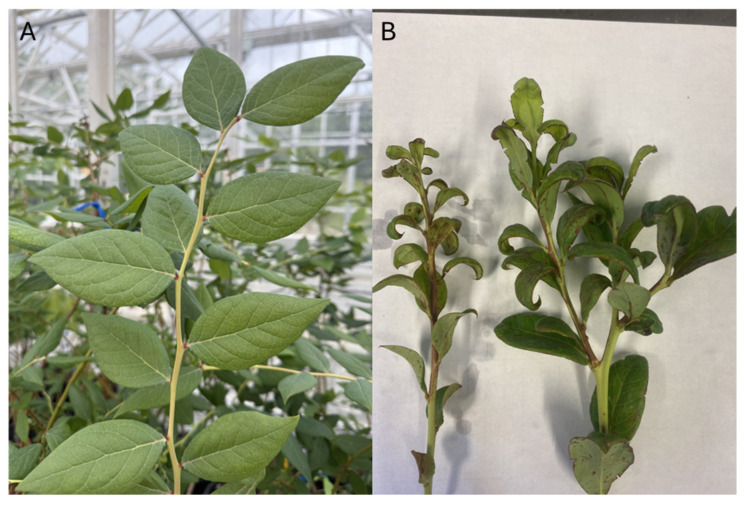
Healthy blueberry twig (**A**) and *S. dorsalis* injury to blueberry twigs and leaves (**B**) (Picture credit: Rosan Adhikari).

*Scirtothrips dorsalis* poses a significant threat to the blueberry crop by feeding on foliage. Although it is not directly impacting the fruit harvest of spring, prolonged infestations can weaken plants, hinder growth, and ultimately reduce yield [18]. *Scirtothrips dorsalis* can also become a serious problem during extended periods of hot and dry weather, typically from June to September. These pests are particularly harmful to young blueberry leaves in the summer and early fall, often starting their attack in late June when the first new leaves appear after pruning [18]. Adult and larval thrips damage plants by puncturing the outer cells of leaves with their mandibles and then inserting a stylet to suck out the contents of the mesophyll tissues [18]. This feeding behavior leads to tissue necrosis and eventually death. Initially, the damage appears as bronzing along the leaf veins and petioles, with leaves gradually curling and becoming distorted (Figure 12). Severe infestations can cause significant defoliation, with the leaves curling extensively before falling off the plant [18]. To manage this pest effectively, two thresholds and sequential sampling plans might be necessary: one for summer to protect young plants from damage and another pre-harvest threshold for older fruit-bearing bushes to prevent yield loss [62]. Detecting an infestation early is crucial for managing this pest effectively. One of the earliest signs of *S. dorsalis* in blueberry fields is the bronzing of new growth [18].

## 7. Management of Thrips in Blueberries

### 7.1. Monitoring

Monitoring thrips is a critical component of effective pest management, aimed at detecting early infestation and guiding control actions before economic damage occurs. Field scouting is one of the primary monitoring methods [63]; growers regularly inspect plants for signs of thrips feeding, such as silvering, scarring, or distorted plant tissue, particularly in young leaves and flower buds, where thrips tend to concentrate. In addition to visual inspections, sticky traps—typically blue, white, or yellow—strategically placed throughout the crop at plant height to capture adult thrips, provide an estimate of population levels [21,64]. When monitored regularly, these traps allow farmers to track population trends over time. However, they often do not accurately represent the actual number of thrips present within flowers, as they are influenced by trap placement, color, and surrounding vegetation [65]. Another key method is direct plant sampling, where parts of the plant (such as leaves or flowers) are shaken or tapped over a tray to dislodge thrips for counting. This method is particularly useful when correlating pest density with injury levels. However, it is labor-intensive, time-consuming, and not always practical for large-scale commercial operations [7,63]. Suction devices, such as aspirators or specialized vacuums, are also used to collect thrips from the plant surface for more precise monitoring but become inefficient when applied to full field monitoring due to labor demands and limited coverage [66]. Degree-day models, which track temperature accumulation to predict thrips development stages, can also be used to help growers time their monitoring efforts more effectively. Nevertheless, their reliability is often compromised by spatial and temporal variability in weather conditions, which can influence thrips development differently across regions and growing seasons [67].

Given these trade-offs, growers and researchers must choose tools based on their monitoring goals, scale of operation, and available resources. For routine scouting, sticky traps can help identify periods of increased activity, while direct sampling or aspirator use may be warranted when assessing injury thresholds. Integrating multiple tools, such as sticky trap data, to trigger direct sampling may offer a more balanced and effective monitoring strategy. There is an opportunity to implement targeted insecticide applications for thrips control in blueberry fields. When thrips populations are high, a first insecticide application can be recommended 7 to 10 days after blooming begins, especially when hot spots have been identified [7]. A second application may be warranted 12 to 16 days after bloom initiation, as this is when hot spots tend to reach their peak population and pose the greatest threat to the crop, potentially causing significant economic loss for the grower [7].

### 7.2. Economic Thresholds

Although economic thresholds (ETs) are referenced as critical tools in IPM to guide decision-making and prevent economic loss [68], well-defined functioning economic thresholds for thrips in blueberries are still largely lacking. This gap is primarily due to the difficulty in establishing consistent relationships between thrips densities and actual yield losses under varying field conditions. As a result, most current guidelines rely on action thresholds, which are based on field observations rather than rigorous economic injury level (EIL) assessments.

Action thresholds are field-observed pest levels used to trigger management actions, whereas economic thresholds are based on quantified pest densities that are expected to cause yield losses justifying the cost of control. For example, an infestation of 2–6 *F. tritici* per cluster of eight individual flowers is often considered indicative of a developing problem, while more than 6 thrips per cluster suggests a severe infestation requiring immediate intervention [57]. For *F. bispinosa*, an action threshold of 100 thrips per white sticky trap (14 cm × 23 cm) per week has been proposed as a trigger for control measures [58].

Thresholds can also vary due to multiple interacting factors, including cultivar-specific susceptibility, climatic conditions, flowering phenology, and the species composition of thrips populations. Rhodes et al. (2012) reported significant variation in fruit injury among blueberry cultivars, but this did not directly correlate with thrips density, suggesting that some cultivars may exhibit greater tolerance to feeding damage [58]. Moreover, species-specific differences in feeding behavior and injury potential further complicate threshold development. For instance, while high numbers of *F. tritici* and *F. bispinosa* may not cause substantial damage, even low populations of *F. occidentalis* can lead to significant fruit scarring [69]. Adding to this complexity, *F. bispinosa* adults are highly mobile and can quickly recolonize treated areas from untreated field edges, potentially making treatments appear ineffective [69]. This mobility, along with the short flowering window in blueberries, makes it difficult to apply conventional thresholds consistently or effectively.

Although action thresholds are used in blueberry production for practical management of thrips, there are currently no widely validated economic thresholds based on direct yield loss data. Developing such thresholds will require further research that accounts for cultivar-specific responses, thrips species biology, and the economic impact of varying levels of infestation.

### 7.3. Cultural Control

Many fruit growers use cultural and physical methods, such as weed control and sanitation, to reduce thrips populations. Basic sanitary practices, such as removing weeds, old plant materials, and debris, have served as the first line of defense against thrips. These actions reduce potential breeding sites and disrupt the life cycle of thrips [70]. After the summer harvest, blueberries are pruned to encourage new growth, which can disrupt *S. dorsalis* populations and potentially prevent their buildup [18]. However, proper disposal of pruned shoots is essential to prevent further infestations, as *S. dorsalis* may spread from the defoliated leaves. Additionally, UV-reflective mulch has proven effective in repelling thrips by interfering with their ability to locate and orient themselves toward host plants. The reflective surface of the mulch confuses the insects, making it harder for them to find and colonize crops, thus offering a non-chemical approach to pest control [71,72].

Kaolin applications have been shown to consistently reduce the population of adult thrips in rabbiteye blueberry by approximately 50% [17]. Applying kaolin over a broader area in blueberry fields could therefore provide effective control of thrips, particularly during critical periods such as peak bloom and the early stages of fruiting when the risk of damage from thrips is at its highest [17]. Kaolin particle barriers could significantly impact the foraging behavior of adult thrips within clusters of flowering and fruiting blueberries. This effect could help manage thrips populations more effectively by altering their movement and reducing their numbers on treated plants [17].

### 7.4. Biological Control

Biological control uses the augmentative release of natural enemies as well as conservation approaches to sustain their abundance and efficiency [73]. The principal biological control agents associated with thrips are minute pirate bugs (*Orius* spp.) and phytoseiid mites (*Amblyseius* spp.) [74]. These small, beneficial predators play a vital role in suppressing thrips populations across agricultural and horticultural systems. Their conservation is a key component of IPM strategies [20]. *Frankliniella* spp. are naturally kept in check by several predators, particularly the minute pirate bug, *Orius insidiosus* (Say) (Hemiptera: Anthocoridae) [69]. *Orius insidiosus* is particularly effective due to its ability to feed on multiple life stages of thrips, including eggs, larvae, and adults, though it shows a strong preference for larvae, which are less mobile and more vulnerable compared to the agile adult thrips [75].

Studies show that even a low predator-to-prey ratio of one *O. insidiosus* to 180 thrips is sufficient to suppress thrips populations, while a higher density of one *O. insidiosus* to 40 thrips can effectively control the infestation [69]. This indicates that even at lower predator densities, *O. insidiosus* can significantly impact thrips populations, reduce their numbers, and minimize damage to crops. When the predator-to-prey ratio is increased, the control becomes more robust, leading to a more thorough reduction in the thrips population [76].

In addition to *O. insidiosus*, a predatory mite, *Amblyseius swirskii*, offers superior control of thrips compared to other predatory mites. Its ease of rearing and ability to be mass-produced have made *A. swirskii* increasingly popular for managing thrips in vegetables, ornamentals, and fruit crops [74,77]. Other commonly used biological control agents include nematodes and certain fungal species. For nematodes, *Steinernema feltiae* is often utilized, while for fungi, *Lecanicillium lecanii*, *Metarhizium brunneum*, *Beauveria bassiana*, and *Isaria fumosorosea* are employed to manage thrips [74].

While these biological control agents have demonstrated potential in various cropping systems, their success largely depends on timing, environmental conditions, and the biology of pests and natural enemies. In the case of blueberry, several factors limit the effectiveness of biological control strategies against *F. tritici* and *F. bispinosa*. These thrips typically arrive in the fields just as the flowers are beginning to open, when there is minimal insect activity in the foliage. Thrips are generally present for only about 20 to 25 days, coinciding with the blueberry flowering period [19]. This short window creates a unique situation for control in blueberries, as it is not sufficient for natural enemies to establish themselves and provide meaningful control of the thrips [19].

Field evaluations have shown that inundative releases of *O. insidiosus* and *Neoseiulus cucumeris*, whether used preventatively or curatively, have not proven significantly effective in managing *F. bispinosa* populations in blueberry fields in Florida [78]. Additionally, adult *F. bispinosa* moves more quickly to the alternative hosts, which could help them evade predators more effectively [37].

To improve the success of biological control in blueberry systems, several strategies can be considered. First, early-season inoculative releases of natural enemies could help establish populations before thrips arrive in large numbers during bloom. Second, implementing banker plant systems, which involve planting non-crop vegetation that supports alternative prey, can help sustain predator populations when thrips densities are low. Ultimately, improving biological control will require a systems-based approach that integrates habitat management, timely release strategies, and conservation biological control.

### 7.5. Chemical Control

While chemical controls are widely used, timing and selectivity are critical for avoiding disruption of beneficial insects and pollinators. Chemical control is one of the most frequently used methods to suppress thrips populations in the blueberry planting systems [79]. Numerous insecticides are registered for managing *F. tritici* in fruits and vegetables. However, it is recommended to delay chemical interventions until action thresholds are met [53]. Additionally, delaying chemical interventions encourages the conservation of natural predators and beneficial insects in the ecosystem, which can also contribute to controlling thrips populations [53].

Among the chemical options, neonicotinoid insecticides, such as imidacloprid and acetamiprid, are commonly used for controlling thrips. Acetamiprid, novaluron, and thiamethoxam were found to significantly reduce the growth rate of thrips populations when compared to malathion [80]. Recent work by Adhikari et al. (2024) showed that insecticides such as acetamiprid, spinetoram, and spinosad effectively reduced adult and larval *F. tritici* populations in rabbiteye and southern highbush blueberries, with efficacy varying by insecticide, thrips life stage, and post-treatment interval [81]. While acetamiprid and spinetoram showed consistent adult suppression across multiple sampling dates, spirotetramat was particularly effective against larvae in early post-treatment stages, emphasizing the importance of life-stage-specific pest management strategies [82].

Similarly, Renkema et al. (2020) evaluated season-long insecticide programs for controlling *F. bispinosa* and *S. dorsalis* in Florida strawberries, finding that spinetoram, acetamiprid, and cyantraniliprole were highly effective against thrips, while organic options spinosad showed moderate efficacy [83]. While efficacious against thrips, neonicotinoids have been linked to negative effects on pollinators and other beneficial insects [80]. Liburd and Arevalo (2019) also indicated that acetamiprid and thiamethoxam were the most toxic insecticides to *O. insidiosus*, with a rapid effect that notably decreased their population within just two hours [19].

In contrast, spinosad, a natural reduced-risk insecticide derived from an actinomycete bacterium, is compatible with natural enemies and currently provides the effective control of *F. tritici*, *F. bispinosa* [84], and *S. dorsalis*. This insecticide is less harmful to *O. insidiosus*, killing only about 30% of the insects after 24 h of exposure. Using reduced-risk insecticides such as spinetoram and spinosad can help conserve *O. insidiosus*, a key natural enemy of thrips, while still providing good suppression of thrips populations [19].

Furthermore, blueberries are highly dependent on pollination for optimal fruit set, making the presence of pollinators, particularly bees, essential during flowering. Improper or poorly timed insecticide applications can negatively impact bee populations, leading to reduced pollination efficiency and ultimately lower fruit yield [85]. These should be applied early in the morning or late in the evening, allowing at least a three-hour drying period before pollinator activity begins. In contrast, insecticides like acetamiprid and tolfenpyrad should be limited to pre-bloom applications to avoid harming pollinators [85].

## 8. Conclusions

Thrips have emerged as significant pests in blueberry production systems, particularly in the Southeastern United States, where *F. tritici*, *F. bispinosa*, and *S. dorsalis* are the most prevalent species. These pests cause economic damage by feeding on floral and vegetative tissues, leading to reduced fruit quality and yield. Thrips’ highly mobile nature, polyphagous behavior, and rapid reproductive capacity allow them to exploit multiple host plants and evade management interventions. Monitoring and identification remain critical for timely and effective management. However, morphological similarities among thrips species complicate field identification, necessitating a combination of morphological and molecular approaches for accurate species confirmation. While some action thresholds are available, rigorously validated economic thresholds based on yield loss data are still lacking. This gap underscores the need for additional research on cultivar-specific injury levels and the economic impact of infestation. Effective management of thrips requires a combination of monitoring techniques, economic threshold assessments, and control techniques, including cultural, physical, biological, and chemical control measures. Monitoring tools such as sticky traps and direct plant sampling are essential for early detection, while cultural practices such as removing plant debris and using UV-reflective mulch help reduce thrips populations. Biological control, particularly with natural enemies including *O. insidiosus* and predatory mites, plays a role, although its efficacy is limited due to the short presence of thrips during the blueberry flowering period. Chemical control remains a cornerstone of thrips management, especially during bloom. However, care must be taken to minimize harm to beneficial insects, particularly pollinators like bees, and predators like *O. insidiosus*. Adhering to pollinator-safe spray timings and selecting insecticides with low non-target toxicity is essential for sustainable management. Managing thrips in blueberries requires a multifaceted approach tailored to species composition, local environmental conditions, and crop phenology. The development of reliable economic thresholds, along with species-specific and environmentally conscious control strategies, will be critical to improving thrips management and minimizing economic losses in commercial blueberry production.

This review provides a comprehensive synthesis of the biology, ecology, and management of the three most economically important thrips species—*F. tritici*, *F. bispinosa*, and *S. dorsalis*—affecting blueberry production in the southeastern United States. While several studies have addressed thrips in blueberries, few have integrated species-specific information on their behavior, host associations, life cycles, and responses to control strategies in the context of early-season production systems. By consolidating this information, our review fills critical knowledge gaps and offers a resource tailored to researchers, extension agents, and blueberry growers in this region.

## 9. Future Directions

Future research should focus on addressing critical knowledge gaps that hinder effective and sustainable management of thrips in blueberry production systems. One key priority is the development of robust, species-specific economic thresholds that directly correlate thrips densities with crop injury and yield loss. These thresholds will provide more precise and economically sound decision-making tools for growers. Additionally, IPM programs should be tailored to the unique biology and ecology of *F. tritici*, *F. bispinosa*, and *S. dorsalis*, incorporating species-specific monitoring techniques, control timings, and management strategies.

Given the importance of pollinators to blueberry production, there is also a pressing need to identify and implement pollinator-friendly insecticide options and application schedules. Future IPM efforts must align pest control interventions with pollination periods to avoid disrupting bee activity. Furthermore, climate change poses new challenges, including altered thrips phenology, expanded geographic range, and increased overwintering success. Developing climate-resilient pest management tools such as real-time degree-day models and predictive risk assessment systems will be essential.

Resistance management is another critical area, particularly considering the frequent use of neonicotinoids and spinosyn-based insecticides. Monitoring resistance development and rotating insecticides with different modes of action should be integrated into IPM programs to prolong the efficacy of available chemical tools. Enhancing the success of biological control through improved release strategies, banker plant systems, and habitat management is equally important, especially given the short window of thrips activity in blueberries. By addressing these areas, future IPM programs can become more adaptive, environmentally sustainable, and resilient to changing pest pressures and production conditions.

## Data Availability

No new data were created or analyzed in this study. Data sharing is not applicable to this article.
